# Electronic Coupling in Nanoscale InAs/GaAs Quantum Dot Pairs Separated by a Thin Ga(Al)As Spacer

**DOI:** 10.1186/s11671-015-0973-5

**Published:** 2015-06-26

**Authors:** Yao Liu, Baolai Liang, Qinglin Guo, Shufang Wang, Guangsheng Fu, Nian Fu, Zhiming M Wang, Yuriy I Mazur, Gregory J Salamo

**Affiliations:** College of Physics Science & Technology, Hebei University, Baoding, 071002 People’s Republic of China; School of Material Science and Engineering, Hebei University of Technology, Tianjin, 300401 People’s Republic of China; Institute of Fundamental and Frontier Sciences, University of Electronic Science and Technology of China, Chengdu, 610054 People’s Republic China; Institute for Nanoscience and Engineering, University of Arkansas, Fayetteville, AR 72701 USA

**Keywords:** Quantum dots, Carrier tunneling, Photoluminescence, Carrier lifetime

## Abstract

The electronic coupling in vertically aligned InAs/GaAs quantum dot (QD) pairs is investigated by photoluminescence (PL) measurements. A thin Al_0.5_Ga_0.5_As barrier greatly changes the energy transfer process and the optical performance of the QD pairs. As a result, the QD PL intensity ratio shows different dependence on the intensity and wavelength of the excitation laser. Time-resolved PL measurements give a carrier tunneling time of 380 ps from the seed layer QDs to the top layer QDs while it elongates to 780 ps after inserting the thin Al_0.5_Ga_0.5_As barrier. These results provide useful information for fabrication and investigation of artificial QD molecules for implementing quantum computation applications.

## Background

Recently, many investigations have addressed the fabrication, characterization, and exploitation of self-assembled InAs/GaAs quantum dot (QD) structures due to their unique properties as well as their great potential for various optoelectronic devices [1–8]. In particular, the quantum coupled InAs/GaAs QD pair structures provide an approach to fabricate artificial QD molecules for implementing quantum computation schemes [9–15]. Generally, the fabrication of quantum coupled InAs/GaAs QD pair structures is implemented by growing two layers of InAs QDs with a thin GaAs spacer to form vertically aligned QD pairs. Such bilayer InAs/GaAs QD structures not only enable tuning of the quantum coupling between InAs/GaAs QDs by adjusting the GaAs spacer thickness but also provide flexibility to independently control the QD density and size as well as to improve QD uniformity [16–19]. These advantages make the vertically aligned InAs/GaAs QD pair structure an interesting choice for achieving artificial QD molecules. Most recently, a low InAs growth rate (<0.02 monolayer (ML)/s) has been adopted by researchers to achieve coupled bilayer InAs/GaAs QD structures emitting above 1.3 μm with a significantly improved homogeneous QD size distribution [20, 21]. A photoluminescence (PL) linewidth as narrow as 10.6 meV has been reported for the bilayer InAs/GaAs QD structure. Despite such significant achievements and potential applications, understanding how the QD pairs are coupled and how their proximity may affect their optical and carrier transfer properties is still the key for both fundamental and applied research. In this work, we aim to manipulate the quantum coupling between InAs QD pairs by inserting a thin Al_0.5_Ga_0.5_As barrier. The effect of the thin Al_0.5_Ga_0.5_As barrier on the carrier transfer inside the vertically aligned QD pairs has been carefully studied by PL and time-resolved PL (TRPL) measurements.

## Methods

The InAs/GaAs bilayer QD structures, consisting of two layers of InAs QDs separated by a 10 nm Ga(Al)As spacer, were grown on semi-insulated GaAs (100) substrates by a solid source molecular beam epitaxy reactor [22]. For sample A, after a 0.5-μm GaAs buffer layer was grown at 600 °C, the substrate temperature was lowered to 530 °C and 2.0 ML of InAs was deposited with a growth rate of 0.013 ML/s to form the seed layer of QDs (SQDs). After 10 s of growth interruption, a 35-ML (10 nm) GaAs spacer was deposited on the top of SQDs. Then the substrate temperature was raised to 600 °C, and the sample was annealed for 8 min. After that, the substrate temperature was lowered to 530 °C again and 2.6 ML of InAs was deposited to form the top layer of QDs (TQDs). The TQDs was capped with 20 nm of GaAs at 530 °C and then an additional 60 nm of GaAs at 600 °C. The growth rate for the spacer and cap layer of GaAs was fixed at 0.5 ML/s. The second QD sample, i.e., sample B, was grown with the same bilayer structure, in which a 15 ML Al_0.5_Ga_0.5_As was sandwiched in the middle of 20 ML GaAs to form the 35-ML Ga(Al)As spacer. For reference, samples with just the SQDs and just the TQDs were grown uncapped for AFM investigation.

## Results and Discussion

**Fig. 1:**
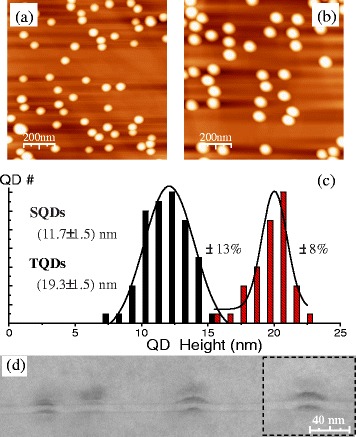
**a** AFM image of the SQDs. **b** AFM image of the TQDs. **c** QD height distribution of the SQDs and TQDs. **d** XTEM image of the sample B

The 1 × 1 μm AFM images of the QDs as well as the QD height distribution are shown in Fig. 1a–c, respectively. The AFM images indicate that SQDs formed by 2.0 ML of InAs have an average diameter of 68.5 ± 6.3 nm and an average height of 11.7 ± 1.5 nm with an areal density of 5.6 × 10^9^ cm^−2^, whereas the TQDs formed by 2.6 ML of InAs have an average diameter of 83.0 ± 6.3 nm and an average height of 19.3 ± 1.5 nm, with an areal density of 5.5 × 10^9^ cm^−2^. The histogram of the QD height distribution shows that both layers of QDs, in particular the TQDs, are larger than usual InAs QDs. Also, the standard deviation (~8 %) of the TQDs average height is much better than that of the SQDs (~13 %). No large incoherent islands are observed on the surface, indicating good sample quality for both QD layers. In our experiments, the very low growth rate (~0.01 ML/s) used for the InAs QDs resulted in the formation of large, homogeneous, high-quality QDs. At the low growth rate, the indium adatoms have a long migration length and thereafter preferentially incorporate into existing QDs to form large islands than forming new small QDs. The long migration length also produces a better size homogeneity of the QDs [20–22]. This is due to the fact that when indium adatoms migrate longer, they have a higher chance of finding a lower energy position in which to be incorporated. This has been observed previously in the AFM of similarly grown QD samples.

The AFM images also show that both layers of QDs have an equal density, indicating the possibility that the QDs are vertically aligned into pairs. The cross-sectional TEM in Fig. 1d confirms this vertical correlation in the QD pairs for both samples. The QD dimensions obtained from the cross-sectional TEM show a diameter of ~28 nm and a height of ~7 nm for the SQDs, whereas a diameter of ~45 nm and a height of ~10 nm for the TQDs. We believe that the 8 min annealing at 600 °C after the initial 35-ML (10 nm) GaAs capping layer makes both SQDs and TQDs become much smaller than the uncapped QDs shown by AFM, although the uncapped QDs likely look larger than their true dimensions due to AFM tip effect. Here, it is also found that the separation between the bottom of TQDs and the tip of the SQDs is only 2~3 nm. Such a thin GaAs barrier will lead to strongly electronic coupling and hence inter-layer carrier transfer from the small SQDs to the large TQDs [23, 24].

**Fig. 2:**
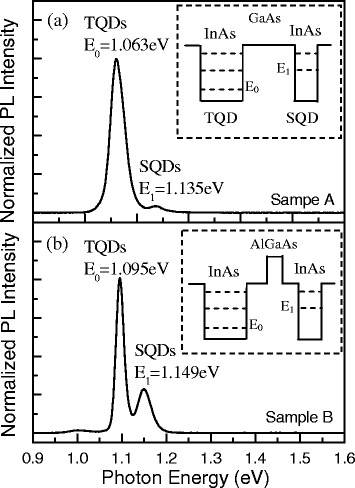
Low-temperature (10 K) PL spectra obtained with a low laser excitation intensity of 30 mW/cm^2^ for (**a**) sample A and (**b**) sample B. The *inset* shows the schematic diagram of the band structure of sample A and sample B, respectively

Carrier transfer within the QD pairs is carefully studied by PL measurements in a variable temperature (10–300 K) closed-cycle cryostat under the excitation of a 532-nm continuous-wave laser. The PL signal is sent to a 0.5-m spectrometer through a 50× objective lens and then detected by a liquid nitrogen-cooled CCD detector array. The PL spectrum at low temperature (10 K) and low excitation intensity (30 mW/cm^2^) shows that either sample A or sample B has a double-peak characteristic. As shown in Fig. 2a for sample A, the peak at *E*_0_ = 1.063 eV is attributed to the electron-hole ground-state transition from TQDs, which exhibited a Gaussian profile and achieved a narrow full-width-at-half-maximum (FWHM) of 28.0 meV. This narrow PL indicates that the top layer of TQDs is very uniform, which is consistent with the results of AFM analysis in Fig. 1. The weak peak at *E*_1_ = 1.135 eV is attributed to the electron-hole ground-state transition from SQDs. The relative small intensity of the SQD PL peak indicates a strong carrier transfer from SQDs to TQDs. Notably, the low-temperature PL spectrum in Fig. 2b from sample B shows that the emission from the SQDs (*E*_1_ = 1.149 eV) is significantly more intense than that from sample A in Fig. 2a. This indicates that the insertion of a thin Al_0.5_Ga_0.5_As barrier has reduced carrier transfer between layers in this sample. In addition, there is a small blue-shift of both peaks of the PL from sample B in comparison with sample A. This is likely due to the change of the confinement potential resulting from the addition of the Al_0.5_Ga_0.5_As barrier [24].

**Fig. 3:**
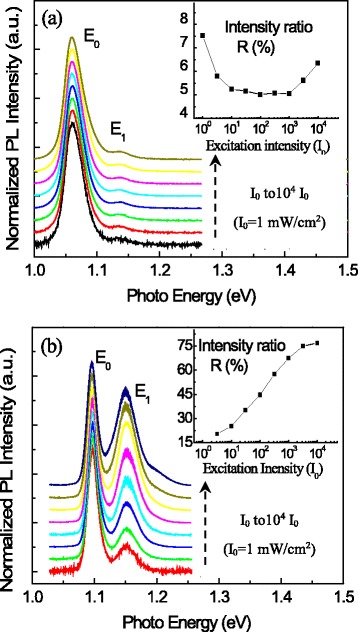
Low-temperature PL spectra of (**a**) sample A and (**b**) sample B as a function of the excitation laser intensity. The *insets* show the PL intensity ratio *R* of the *E*_1_ emission to the *E*_0_ emission (*R* = *E*_1_ emission/*E*_0_ emission × 100 %) as a function of the excitation laser intensity

The PL spectra are presented in Fig. 3 as a function of the laser excitation intensity for both samples. For convenience, each spectrum is normalized and shifted up. The insets give the PL peak intensity ratio *R* between emission from SQDs and TQDs. As shown here, these two samples have remarkably different excitation intensity dependencies. For sample A, the ratio *R* initially decreases as the excitation intensity increases. It means that, due to strong carrier transfer from SQDs to TQDs, the photon-generated carriers even initially relax into SQDs, most of them will finally go to the TQDs through tunneling to obtain optical recombination. Therefore, the intensity of the *E*_1_ emission increased slower than that of the *E*_0_ emission. After the *E*_0_ emission from TQDs becomes saturated at ~10^3^*I*_0_ (*I*_0_ = 1 mW/cm^2^), the ratio *R* reaches a minimum then increases. In other words, after the *E*_0_ emission from TQDs gets a saturation, the carrier transfer from the SQDs to TQDs reduces; thereafter, the carriers inside the SQDs would have more opportunity to get an optical recombination rather than transferring to TQDs. This excitation dependence of QD PL intensity ratio is a feature of the InAs QD pairs with strong electronic coupling. However, for sample B with the AlGaAs barrier, *R* increases throughout the entire range of excitation intensity dependence. The variation of *R* indicates that there is still carrier transfer from SQDs to TQDs, although this carrier transfer is not as strong as that in sample A. It leads to that the intensity of the *E*_1_ emission increased faster than that of the *E*_0_ emission.

**Fig. 4:**
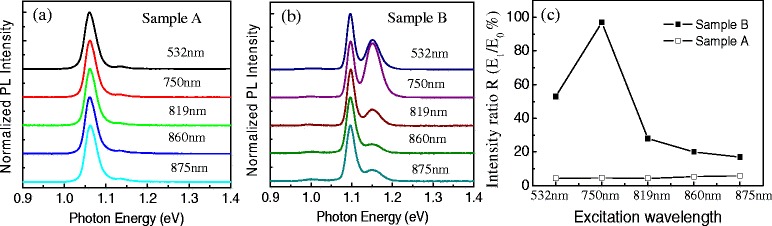
Low-temperature PL spectra of (**a**) sample A and (**b**) sample B measured with different excitation laser wavelengths. **c** The PL peak intensity ratio *R* as a function of excitation laser wavelength

We then measure PL spectra at *T* = 10 K by using different laser wavelengths but the same intensity (100 mW/cm^2^) to excite carriers at different layers inside the samples. Each spectrum is normalized and shifted up. As in Fig. 4a, the PL spectral profiles of sample A change very little using different excitation wavelengths and *R* remains constant as shown in Fig. 4c. This is due to the strong carrier tunneling in this sample. No matter the carriers are generated in the GaAs matrix or in the wetting layers, and even if the photon-generated carriers are initially collected by the SQDs, most of them still go to TQDs and recombine there. However, the PL spectra of sample B show different features. The relative peak intensity at *E*_1_ = 1.149 eV from SQDs changes widely with respect to the excitation wavelength. We believe that for a laser with photon energy above GaAs bandgap, the photon-excited carriers are predominantly generated on the substrate side of the GaAs layers and, thus, favored to fill the ground-state energy level of the SQDs while the AlGaAs layer could act as a carrier barrier to make carrier transfer not efficient as in sample A. In particular, the peak at *E*_1_ = 1.149 eV appears to exhibit a pseudo-resonant enhancement when the excitation wavelength is closed to GaAs bandgap. For the wetting layer (excitation laser wavelength *λ* = 860 nm) or sub-wetting layer excitation (excitation laser wavelength *λ* = 875 nm), *R* does not vary much.

**Fig. 5:**
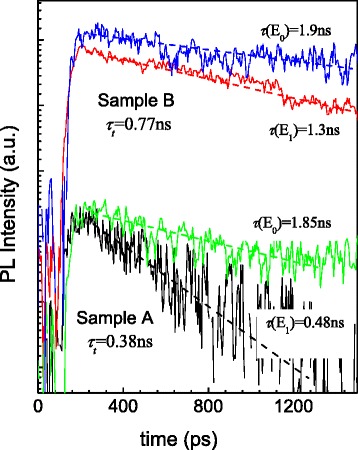
TRPL results for the SQDs and TQDs for both sample A and sample B

The carrier coupling is further investigated through the temporal decay behavior for both samples at 10 K. For TRPL experiments, the samples are excited by a Ti:Sapphire mode-locked laser (780 nm, 78 MHz, 2.7 ps) and a C5680 Hamamatsu streak camera with the infrared-enhanced photocathode is used as detection system. The decay curves are measured with excitation intensity of ~4 × 10^7^ photons/pulse for the QDs of both the seed and the top layers. As shown in Fig. 5, for both samples, the SQDs have a faster decay than the TQDs. This indicates that there is carrier tunneling between the two layers of QDs. However, the SQDs of sample A have a very short decay time (*τ*_(*E*1)_ = 0.48 ns) in comparison with the TQDs(τ_(E0)_ = 1.85 ns), while the SQDs of the sample B have a relatively long decay time (*τ*_(*E*1)_ = 1.3 ns). The TRPL indicates the existence of a strong competition between tunneling and radiative recombination for the carriers inside the SQDs. Taking a simple model of the SQDs and the TQDs forming a three-level system, the measured PL decay time *τ*_*t*_ for the SQDs is approximately given by:

1 $$ {\tau}_{\mathrm{PL}}={\tau}_s\cdot \frac{\tau_t}{\tau_s+{\tau}_t} $$

where *τ*_*t*_ is the carrier tunneling time from SQDs to TQDs and *τ*_*S*_ is the radiative lifetime of carriers in SQDs [25, 26]. From the experimental data shown in Fig. 5 and using Eq. (1), we estimate the carrier tunneling time *τ*_*t*_ to be 380 ps for sample A and 780 ps for sample B. The insertion of the AlGaAs barrier nearly doubles the carrier tunneling time in our experiments.

We also calculate the carrier tunneling time for both samples using a modified form of the semiclassical Wentzel–Kramers–Brillouin (WKB) approximation [26–28]. In this approximation, *τ* is given by:

2 $$ \tau =\frac{1}{vT}=\frac{m^{*}}{8\hslash}\cdot \frac{\rho_0^4}{\kappa_0^2{K}_0^3}\cdot \omega \cdot \exp \left(2{\kappa}_0b\right) $$

here $\nu =\frac{\hslash {\kappa}_0}{2{m}^{*}\omega }$ is the quasi-classical collision frequency of the carriers, $T=\frac{16{\kappa_0}^2{K_0}^2}{{\rho_0}^4}\cdot \exp \left(2{\upkappa}_0\mathrm{b}\right)$ is the transmissivity of electrons passing through the potential barrier and ${\kappa}_0=\frac{\sqrt{2{m}^{*}\left({V}_0-{E}_0\right)}}{\hslash }$, ${K}_0=\frac{\sqrt{2{m}^{*}{E}_0}}{\hslash }$, and ${\rho}_0=\frac{\sqrt{2{m}^{*}{V}_0}}{\hslash }$. Also here, *ω* is the QD height, *b* is the thickness of barriers, *V*_0_ is the height of barriers, and *E*_0_ is intrinsic energy of the bound states. Using Eq. (2), the carrier tunneling time is determined to be *τ*_*A*_ = 0.31 ns for sample A and *τ*_*B*_ = 0.68 ns for sample B, which correspond well with the experimental results.

## Conclusions

In summary, vertical energy transfer for InAs/GaAs QD pair structures with and without an AlGaAs barrier was compared. Low-temperature PL measurements show that the QD peaks shift to the blue and the relative PL intensities of the two QD layers change as a result of adding the AlGaAs barrier. In addition, the dependencies of the intensity ratios on excitation laser intensity and wavelength are very different with the AlGaAs barrier. TRPL measurements give a carrier tunneling time from the seed layer QDs to the top layer QDs of 380 ps. However, the carrier tunneling time increases to 780 ps due to the Al_0.5_Ga_0.5_As barrier. These results help in the understanding of carrier tunneling and the manipulation of energy transfer within InAs quantum dot molecules. This provides useful information for the fabrication of artificial QD molecules in order to implement quantum computing architectures.
